# Drug-Induced Thrombocytopenia following a Transvaginal Oocyte Retrieval for In Vitro Fertilization

**DOI:** 10.1155/2015/890610

**Published:** 2015-02-25

**Authors:** Ioanna A. Comstock, Michelle Longmire, Richard H. Aster, Amin A. Milki

**Affiliations:** ^1^Stanford University Medical Center, 900 Welch Road, Suite 20, Palo Alto, CA 94304, USA; ^2^Stanford University Medical Center, 450 Broadway Street, Redwood City, CA 94063, USA; ^3^BloodCenter of Wisconsin, Medical College of Wisconsin, P.O. Box 2178, Milwaukee, WI 53201, USA; ^4^Stanford University Medical Center, 900 Welch Road, Suite 350 MC 5800, Palo Alto, CA 94304, USA

## Abstract

Drug-induced immune thrombocytopenia has been associated with hundreds of medications and can lead to devastating consequences for the patient. We present a case of a healthy 33-year-old female undergoing in vitro fertilization who developed a severe drug-induced thrombocytopenia, petechiae, and a large hemoperitoneum after receiving Cefazolin antibiotic prophylaxis for a transvaginal oocyte retrieval. The patient was admitted to the intensive care unit for resuscitation with blood products. The presence of drug-dependent platelet antibodies to Cefazolin was confirmed serologically.

## 1. Introduction

Since its introduction in 1985 [1], transvaginal ultrasound guided oocyte retrieval (TVOR) has become the gold standard for oocyte aspiration in IVF therapy worldwide. Despite its simplicity, effectiveness, and widespread use, there has been limited data regarding serious complications arising from this procedure. Although rare, the most common complications are hemorrhage, inadvertent injury of nearby pelvic organs/vessels, and pelvic infection [2]. The incidence of pelvic infections reported in the literature is 0.3 to 0.6% [2]. Despite this low incidence, fertility practices are treating patients with prophylactic antibiotics in addition to vaginal preparation for the procedure. There is no general consensus with regard to routine antibiotic prophylaxis, selected antibiotic use for high risk patients, type of antibiotic, or timing of administration. In our practice, we routinely administer a cephalosporin prior to vaginal puncture unless the patient has an allergy. If that is the case, an alternative antibiotic is chosen.

In this report, we describe a case of drug-induced thrombocytopenia causing a large hemoperitoneum requiring transfusion of blood products after administration of Cefazolin for antibiotic prophylaxis during a transvaginal oocyte retrieval.

## 2. Case

A 33-year-old woman presented to our assisted reproduction facility for treatment of male factor infertility and embryo cryopreservation. A baseline CBC was obtained and showed a hemoglobin of 13.6, hematocrit of 41.8, MCV of 101.8, and platelet count of 145,000.

An antagonist protocol was used for controlled ovarian hyperstimulation (COH) using FSH and HMG. TVOR was performed under monitored anesthesia care (MAC) using Propofol and Meperidine. The patient received 1 gram of Cefazolin for antibiotic prophylaxis and Ondansetron 4 mg for postoperative nausea.

Upon completion of the procedure, a transvaginal ultrasound showed minimal free fluid in the pelvis. The patient recovered well after the procedure and was discharged in stable condition. Her vital signs were within normal limits and she had minimal discomfort postoperatively.

Our office policy is that patients recover at home postoperatively until the following morning. However, the patient felt well enough to return to work after lunch that afternoon. She worked for approximately 2 hours when she experienced two separate vasovagal episodes during which she did not lose consciousness. Approximately six hours postoperatively, she noted petechiae developing on her arms, legs, back, and chest and called an ambulance to be taken to the emergency department. Upon arrival in the ED, she was hemodynamically stable with a normal physical exam aside from the petechiae. Her abdomen was mildly tender consistent with her recent procedure and ovarian stimulation. However, there was no evidence of abdominal distention at initial presentation.

A CBC was drawn and showed hemoglobin of 11.4, hematocrit of 34, and platelet count of 2,000. Her INR was 1.1, prothrombin time 13.8, fibrinogen 261, D-dimer 6677, LDH 167, reticulocyte count 1.8%, and troponin was negative. A repeat CBC showed hemoglobin of 9.2, hematocrit of 27.5, and platelet count of 4,000.

While being monitored in the ED, the patient's vital signs remained stable but her abdomen became clearly more distended. A transabdominal ultrasound done at the bedside revealed bilateral enlarged ovaries consistent with recent controlled ovarian hyperstimulation. She did have a moderate amount of free fluid in the mid to upper abdomen that presumably developed during her time in the ED, likely secondary to her thrombocytopenia and oozing from her ovarian puncture sites. There was very little suspicion for a surgical complication leading to vessel injury or active bleeding from the ovary given the late onset of her symptoms and the unusually severe thrombocytopenia that is unlikely to result from a consumptive coagulopathy.

Hematology was consulted to treat her thrombocytopenia. At this point their differential diagnosis included idiopathic (autoimmune) thrombocytopenia (ITP), drug-induced thrombocytopenia, consumptive coagulopathy, anti-phospholipid antibody syndrome, disseminated intravascular coagulation (DIC), and thrombotic thrombocytopenic purpura (TTP).

Her initial labs did not support hemolysis and a peripheral blood smear showed normal RBCs, WBCs, but absent platelets.

The patient was transferred to the intensive care unit for continued resuscitation with blood products and further workup of the thrombocytopenia. A CT of the abdomen and pelvis showed a moderate hemoperitoneum but no active arterial extravasation. The ovaries were enlarged bilaterally with multiple small foci of gas secondary to recent retrieval. In the ICU, the patient received 4 units of packed red blood cells, 4 units of platelets, and 2 units of fresh frozen plasma. There was gradual improvement in her platelet count overnight with the administration of blood products. A CBC was checked every 4 hours the following day and her counts remained stable—hemoglobin 10.6–10.9, hematocrit 30.4–32, and platelets 121,000–140,000. On postoperative day 2, the patient remained hemodynamically stable and was feeling better. She was discharged home with the plan to follow-up with hematology as an outpatient for a further thrombocytopenia workup.

The patient independently investigated the possibility of a drug-induced thrombocytopenia by contacting Dr. Aster who had published a review article in the New England Journal of Medicine [3] regarding this disorder. A blood sample was sent to the BloodCenter of Wisconsin for testing in a flow cytometric assay [4]. The patient was found to have strong Cefazolin-dependent platelet-reactive antibodies (both IgM and IgG) confirming the diagnosis of a Cefazolin-induced immune thrombocytopenia. Upon further investigation, the patient recalled that she received Cefazolin in the past for a dental procedure. There were no antibodies specific for Ondansetron or Meperidine.

The patient underwent a second IVF cycle three months later. TVOR was performed with Fentanyl 75 mcg and no prophylactic antibiotics. A transvaginal ultrasound performed immediately postoperatively showed minimal free pelvic fluid. This was repeated one hour later and was unchanged. The patient's vital signs were normal and a CBC drawn in the recovery room showed a hemoglobin of 13, hematocrit of 37, and platelet count of 157,000. Serial CBCs were drawn at 4 hours and 8 hours postoperatively and remained stable.

## 3. Discussion

Despite a very low incidence of pelvic infection occurring after TVOR, prophylactic administration of antibiotics is commonly used in fertility clinic practices. It is probable that for the majority of the time a cephalosporin is given preoperatively to a patient undergoing a clean-contaminated procedure such as oocyte retrieval. Among the other common complications associated with this procedure, it is important for a clinician to consider medication reactions both preoperatively and postoperatively while treating patients undergoing in vitro fertilization. Usually, 5–7 days of exposure to a medication is required to induce sensitivity unless there has been prior exposure, as in the patient described here.

Drug-induced thrombocytopenia (DIT) can be triggered by many medications, including antimicrobials, histamine receptor antagonists, and chemotherapeutic agents [3]. A literature search revealed only one prior report of possible Cefazolin-induced thrombocytopenia, a case not confirmed by serologic testing that had associated neutropenia [5]. Several theories have been proposed to explain the pathogenesis of this disorder depending on the type of drug involved [6]. Penicillins and cephalosporins can act as haptens, small molecules that are not themselves immunogenic but can lead to a hapten-specific immune response when attached to a larger carrier protein. It has been suggested that this mechanism may account for immune hemolysis and thrombocytopenia seen in some patients sensitive to this class of compounds [7]. However, alternative mechanisms have been proposed [3].

The incidence of DIT is not well known but is estimated to occur in at least 10 cases per million people per year [8]. Patients with this disorder typically present with petechiae, bruising, and epistaxis 5 to 10 days after taking the causative agent for approximately one week. Occasionally, these patients can present with more severe symptoms such as purpura, bleeding from the gingiva or nose, or gastrointestinal or urinary tract bleeding [3]. The diagnosis of DIT should certainly be considered if patients present with these symptoms and have otherwise unexplained severe thrombocytopenia (platelet count < 20,000).

The most important treatment of these patients is to stop the causative agent. Platelet counts will typically begin to recover within 1-2 days [3]. However, patients with severe thrombocytopenia may require platelet transfusions and other supportive measures depending on their condition, as seen in our patient. Patients strongly suspected of having DIT should also undergo laboratory testing to establish a diagnosis of DIT and identify the drug that led to the reaction [6]. It is extremely important that the patient avoids any future exposure to the drug.

Antibiotics are routinely prescribed and viewed by most prescribers to be fairly benign. However, their potential adverse effects are underappreciated and should be considered by clinicians. We report, to our knowledge, the first case of severe, isolated thrombocytopenia after a transvaginal oocyte retrieval for IVF secondary to a Cefazolin-induced immune reaction.

Given the frequency that this procedure is performed worldwide and likely with antibiotic prophylaxis, it is important that clinicians are aware of this condition and the medications that can be associated with it. It is important to consider the establishment of guidelines regarding antibiotic prophylaxis in IVF patients since the administration of these medications is not without risk and the incidence of pelvic infection is low in patients who do not have risk factors. Additionally, it may be beneficial to request that patients remain geographically local to their IVF clinic for the first 24 hours postoperatively to monitor them for such unforeseen complications even when the index of suspicion is extremely low, such as in this case.
